# User Satisfaction Evaluation of the EHR4CR Query Builder: A Multisite Patient Count Cohort System

**DOI:** 10.1155/2015/801436

**Published:** 2015-10-11

**Authors:** Iñaki Soto-Rey, Aurèle N'Dja, James Cunningham, Axel Newe, Benjamin Trinczek, Caroline Lafitte, Brita Sedlmayr, Fleur Fritz

**Affiliations:** ^1^Institute of Medical Informatics, University of Münster, Albert-Schweitzer-Campus 1/A11, 48149 Münster, Germany; ^2^Clinical Sciences & Operations Platform, Sanofi R&D, 1 avenue Pierre Brossolette, 91385 Chilly-Mazarin Cedex, France; ^3^Center for Health Informatics, University of Manchester, Oxford Road, Manchester M13 9NR, UK; ^4^Chair of Medical Informatics, Friedrich-Alexander-University Erlangen-Nuremberg, 91058 Erlangen, Germany

## Abstract

The Electronic Health Records for Clinical Research (EHR4CR) project aims to develop services and technology for the leverage reuse of Electronic Health Records with the purpose of improving the efficiency of clinical research processes. A pilot program was implemented to generate evidence of the value of using the EHR4CR platform. The user acceptance of the platform is a key success factor in driving the adoption of the EHR4CR platform; thus, it was decided to evaluate the user satisfaction. In this paper, we present the results of a user satisfaction evaluation for the EHR4CR multisite patient count cohort system. This study examined the ability of testers (*n* = 22 and *n* = 16 from 5 countries) to perform three main tasks (around 20 minutes per task), after a 30-minute period of self-training. The System Usability Scale score obtained was 55.83 (SD: 15.37), indicating a moderate user satisfaction. The responses to an additional satisfaction questionnaire were positive about the design of the interface and the required procedure to design a query. Nevertheless, the most complex of the three tasks proposed in this test was rated as difficult, indicating a need to improve the system regarding complicated queries.

## 1. Background

Clinical trials (CTs) are essential to assess the effectiveness and safety of new treatments and procedures. The cost and complexity of CTs have increased in the last decades [1] and initial budgets are often readjusted upwards due to recruitment rates not being met [2] and costly protocol amendments [3].

Optimized protocol designs have proven to be essential in avoiding such issues and ensuring CT success [4]. Study protocol design is the first step in every CT, in which the purpose and detailed methods needed to carry out a certain CT are established. Current protocol design processes include interaction with clinicians located at clinical research institutions, who give their expertise on fundamental matters of protocol design, such as the viability of the trial and the number of possible participants at their site. The responses given by clinicians to these questions are usually obtained through electronic or paper based feasibility assessments, an often slow and cumbersome process, seldom supported by efficient electronic systems. Furthermore, responses from the clinicians are in the majority of the cases based on subjective experience rather than on historical evidence [5].

**Figure 1:**
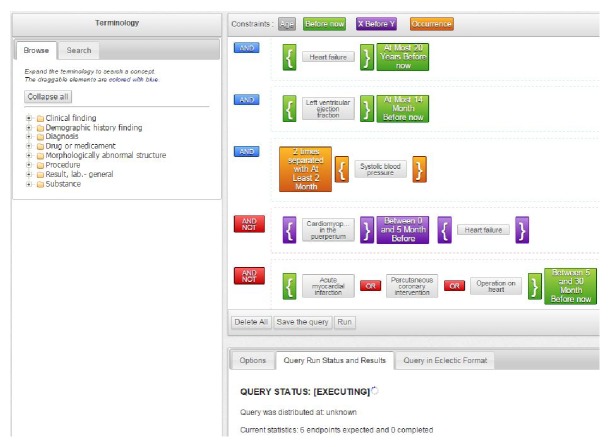
EHR4CR Query Builder. Example of a query built using the EHR4CR QB. The terminology services can be found on the left part of the image and the selected elements and logic on the right part. On the right bottom corner, the user can see the status of the query, the options for each criterion, and the visualisation of the EC in human readable language.

Some initiatives are trying to improve the design of study protocols through the reuse of Electronic Health Records (EHRs) to automate certain process steps. For example, the Shared Health Research Information Network (SHRINE) provides a query tool for open source Informatics for Integrating Biology and the Bedside (i2b2) clinical data repositories [6]. Other examples of the reuse of clinical data to support clinical research include the feasibility platform for stroke studies (FePASS), an open access online web-system that allows users to obtain eligible patient counts for stroke trials based on user-defined eligibility criteria (EC) [7] and the Feasibility Assessment and Recruitment System for Improving Trial Efficiency (FARSITE) tool [8]. These systems often have limitations such as reduced number of temporal constraints available or single source compatibility [9]. Several electronic systems provide support for different process steps of CTs, especially for the case report form completion and the serious adverse event reporting, but there is a need for a single platform that covers a broader variety of the CT process steps [10]. Due to these reasons and limitations, the Innovative Medicines Initiative (IMI) started the Electronic Health Records for Clinical Research (EHR4CR) project (http://www.ehr4cr.eu/) in 2010. The EHR4CR project aims to support the CT steps of protocol feasibility (PF), patient identification and recruitment (PIR), clinical trial execution, and serious adverse event reporting. The EHR4CR technological platform currently supports the PF through the EHR4CR Query Builder (QB), a web-based Java platform with a drag and drop graphic interface (see Figure 1) that allows users to design queries based on CT inclusion and exclusion criteria, send these to specific systems at sites from countries initially across Europe, and automatically obtain, within minutes, the objective number of patients per site matching the given criteria. The system preserves the anonymity of patient data through the shift of dates, fuzzing of low counts, and providing only patient counts (PF) or pseudonymised patient names and identifiers (PIR).

The EHR4CR QB contains a central terminology service (see Figure 1) with several hundred elements from the most important medical classifications and terminologies, as well as all the temporal constrains and Boolean logic needed to build feasibility queries [11]. This system is intended to complement contact with clinicians in the protocol design process by providing reliable quantitative data about availability of patient population in dedicated sites [5]. The EHR4CR QB is also being reused in a PIR scenario of the project, in which the EHR4CR QB allows users located at the clinical sites to identify eligible patient candidates for their potential enrolment in a certain CT if confirmed eligible.

In order to support the EHR4CR platform rollout, the reliability, the usability, and the user- friendliness of the system need to be ensured. In this context, usability is understood as “the extent to which a product can be used by specified users to achieve specified goals with effectiveness, efficiency, and satisfaction in a specified context of use” as defined by ISO 9241-11 [12]. For a company interested in becoming users of the system, it is also essential to know what the learning period for the platform is and what the best methods of obtaining user expertise are.

Several tests have demonstrated the reliability of the EHR4CR source code and the algorithm that calculates the patient counts [9]. In a recent evaluation, the effectiveness and the efficacy of the feasibility process using the EHR4CR QB compared with traditional methods were assessed [13]. However, other systems have been proven to be accurate and effective, while the final software was not usable due to its lack of user-friendliness [14]. Thus, there is a need for a user satisfaction evaluation to ensure that the system fits the user needs and an estimation of the training required for the use of the EHR4CR QB in a production environment.

The objectives of this research are therefore to evaluate the user satisfaction of the EHR4CR QB and to assess whether the training material provided is enough to reach an optimal use of the system.

## 2. Methods

### 2.1. Study Design

According to Harris et al. [15], the study design of this evaluation is quasi-experimental without control groups, in which the participants first experience the intervention (here: training of platform), followed by the observation of the outcome (here: suitability of the training based on success of tasks). Since the target population (end users of the EHR4CR QB) did not represent a large number of candidates within the population source, a small sample size was chosen for this evaluation. Thus, a comparison between groups was not considered. To exclude potential bias, an observation-intervention-observation design has been ruled out as well. The study preserved the anonymity of the test persons and it was approved by the Ethics Committee of Münster (Germany).

### 2.2. Participants

This evaluation was performed in two iterative rounds with professionals familiar with the feasibility domain of clinical research from two different backgrounds: pharmaceutical industry and academia. Representatives of each, involved in the EHR4CR project, were asked to raise awareness among their colleagues. Participants were considered eligible if they (1) had experience in feasibility studies and/or were feasibility managers, (2) did not already know the EHR4CR QB, (3) worked for one of the project partners, and (4) initially agreed on taking the necessary time, answering the usability questionnaire, and recording their computer screen. These potential participants then received a detailed description of the goal, design, overall tasks, tools utilized, data protection, and method of anonymisation of user data via email. They were informed that the participation in this evaluation is completely voluntarily and could be aborted at any time without any consequences.

In the first round, a sample of 22 testers participated, 16 of them belonged to the European Federation of Pharmaceutical Industries and Associations (EFPIA) (http://www.efpia.eu/). The other six participants from academia were a mix of physicians and experts in ergonomics and evaluation of human-machine interfaces and interactions. The pharmaceutical companies represented were Amgen, AstraZeneca, Bayer, GlaxoSmithKline, Lilly, Novartis, and Sanofi. The academic institutions were the Georges-Pompidou European Hospital (HEGP) in Paris and the Evalab from the University Hospitals of Geneva (HUG). All of the institutions (both from academia and from the industry) were members of the EHR4CR consortium. This round was a pretest designed to test the functionality of the system and the study setup and to allow the evaluation team to fix technical issues that users might encounter.

The second round was performed by EFPIA partners (Amgen, Bayer, GlaxoSmithKline, Lilly, and Novartis) only. A sample of 22 completely new testers, familiar with the feasibility domain of clinical research, was recruited, of which 16 participated in this evaluation. For the six other participants, the principal reason for withdrawing was due to scheduling issues. During this round, data for the evaluation was collected.

### 2.3. Material

A training manual containing 20 pages and an 8-minute-long video produced by the evaluation team were provided to the testers on the day of the evaluation via email. The video was an introduction of the EHR4CR QB demonstrating how to create and execute a query. The training manual produced for the evaluation covered the whole procedure giving step by step details (with illustrating pictures) from connecting to the EHR4CR QB to visualizing the results obtained by the system. The time needed to read it was approximately 20 minutes. Together with the manual, a test instructions document was provided to the participants. These instructions were in the format of a test script that users had to follow to perform the evaluation.

To capture data about the quality of the testing material, the performance of the EHR4CR QB, and the success rate, the testers were asked to take screenshots of their screen after the completion and execution of each query during the testing process. Furthermore, to collect testers' feedback (in terms of interface, ergonomics, and usability of the system) and to assess their satisfaction, a self-hosted installation of the web-based open source survey tool LimeSurvey [16] was utilized to conduct a usability survey by means of a questionnaire. Thus, during the testing, participants were asked to complete the questionnaire after finishing (or cancelling) each task to assess each respective part of the test. They were also asked to rate the training provided prior to testing. This method was meant to collect their immediate impression on the EHR4CR QB and allowed the evaluation team to assess whether the training was sufficient or not.

The questionnaire was based on the System Usability Scale (SUS) [17]. Since the SUS did not cover all objectives of this evaluation, it was enhanced under guidance of an experienced psychologist and usability expert. It comprised 4 parts:

- Part A (posttask assessment) was intended to be filled in directly after execution of each task, separately for each task. It comprised 6 questions.
- Part B (usability and acceptance of the QB) had to be filled in after all tasks were completed. It comprised the 13 questions of the SUS with two additional open questions.
- Part C (suitability of the training) also had to be filled in after all tasks were completed. It comprised 9 questions.
- The questionnaire was complemented by Part D (background information) to collect demographic information. It comprised 10 questions.

A dedicated section in the questionnaire was created for the testers to add their screenshots into the survey.

A complete version of the questionnaire is available as additional file (see Supplementary Material available online at http://dx.doi.org/10.1155/2015/801436).

The evaluation was performed on the EHR4CR QB, accessible through the Internet. The URL to the EHR4CR QB was provided by email.

### 2.4. Study Flow

This evaluation was conducted across five countries (France, Germany, UK, Spain, and Switzerland) and performed at the usual workplace of the participants to keep a familiar environment and allow them to focus on the EHR4CR QB and assess the system without external influence.

The evaluation occurred within a one-week period (from December 1 to December 5, 2014). At the beginning of the evaluation, anonymous IDs were randomly assigned to the testers in order to preserve their privacy. Another email was sent out to the testers containing the training material. The EHR4CR QB was made accessible at the same time, and anonymized login credentials were provided. The testers were asked to conduct the self-teaching for about 30 minutes on “how to use the EHR4CR QB” with the instruction manual and the demonstration video. They first had to read the instruction manual to get the necessary level of knowledge before starting to use the EHR4CR QB. Following this, they had to watch the 8-minute demonstration video which was a complement to illustrate the purpose of the manual.

All users then performed platform testing following the same test instructions through three predefined tasks for about one hour and a half. Each task consisted in building a query (a set of inclusion and exclusion criteria) and then running the query against endpoints located at different partner hospitals. The aim was to retrieve patient counts corresponding to the predefined set of criteria. The queries were designed to increase the difficulty in a progressive way in order to capture all functionality of the system (see Table 1). Thus, the first query contained only two criteria to construct (gender + age). The purpose here was to familiarize testers with the engine, familiarizing them with basic steps as an introduction. The second query was more representative of what experts face in early stages of protocol design. Its construction was based on real study design queries and constructed by feasibility experts from within the EHR4CR project. The third query was designed to explore all functionalities of the system as the user had to use different temporal constraints and find several inclusion and exclusion criteria in the terminology engine (see Table 1).

### 2.5. Data Analysis

Based on the responses to the open-ended questions, categories were defined and the responses assigned accordingly. Usability issues mentioned in open-ended questions of questionnaire part A were additionally ranked by a system expert according to their importance. The answers to closed questions of the questionnaire were translated into a numerical scale from 1 (strongly disagree) to 5 (strongly agree). For descriptive analysis, mean scores and standard deviations were calculated. Cases with missing values were deleted listwise. The calculation of the SUS score was based on Brooke's standard scoring method [17]. All values were scaled from 0 to 4, summed up per user, and multiplied by 2.5. This converts the range of possible values from 0 to 100 and allows the values to be compared to a grading scale. Wilcoxon rank sum test was applied for statistically comparing questionnaire results between items. For analyzing if there are groups of relatively homogeneous answers in the SUS results, a cluster analysis was calculated. At first, Ward's method was used to assess the possible number of clusters. Then, *K* Means Clustering was run with a chosen optimum number to place all the cases. To evaluate whether demographic variables have an influence on the questionnaire results, Pearson correlation was applied. The significance level used for testing was 0.05. All statistics were calculated with the software SPSS 22.0.

### 2.6. Task Success Analysis

Screenshots with the results from the query construction in ECLECTIC language [18] and the query execution results were manually reviewed and a classification of the task completion and success per user were built, indicating three different levels of success (success, failure, and partial success) based on similar studies [19]. A successful task was defined as follows. The user was able to complete the creation and execution of the query that matched the task goal, and this one contains exactly the same EC as the one built by an EHR4CR QB expert (and from whom the task was defined). A partial success meant that the tester was able to create the query but this one contained two or less minor errors (e.g., wrong use of the temporal constraints) or one severe mistake (e.g., wrong use of the EC). A failure meant that the tester committed more than one medium or two minor mistakes.

## 3. Results

### 3.1. Participant Characteristics

16 managers and specialists with balanced gender and an average work experience of 3 years (SD: 1.680) participated in the second round of the evaluation study. Half of them (*n* = 9) judged their experience with feasibility studies as high; about 80% of the participants (*n* = 13) had not used similar systems in the past. The majority of the participants (*n* = 14) had good and excellent computer skills. The full sample characteristics are presented in Table 2.

### 3.2. Questionnaire Results

#### 3.2.1. Perceived Task Difficulty and Satisfaction

Across the three tasks of the study task 1 (mean: 3.94, SD: 0.929) and task 2 (mean: 3.75, SD: 1.000) on average were rated as “somewhat easy”; task 3 was judged as “neutral” (mean: 2.63, SD: 1.088). Overall, participants were satisfied with the ease of completing the tasks (task 1: 3.81, SD: 0.911; task 2: 4.06, SD: 0.575) or “neutral” in this regard (task 3: 2.75, SD: 0.858). Satisfaction with the amount of time it took to complete tasks was “rather high” for task 1 (mean: 3.88, SD: 0.885) and task 2 (mean: 3.75, SD: 0.856) and rated as “neutral” for task 3 (mean: 2.94, SD: 0.680). Likewise, satisfaction with functionality provided when completing the tasks was judged as “rather high” (task 1: 3.69, SD: 0.873; task 2: 3.81, SD: 0.655) or “neutral” (task 3: 2.88, SD: 0.957). Significance testing with Wilcoxon rank sum test revealed that all differences of judgements between tasks 1 and 3, respectively, and tasks 2 and 3 were significant (see Table 3).

The analysis of open-ended questions still identified shortcomings of the EHR4CR QB; for example, there was no ability to execute results for all countries, the sequence of building a query was not clear (systems seemed to require it in reverse), there was a lack of system feedback when saving the query, and specific values of criteria were not directly visible because this information required scrolling. Expert review revealed that the majority of these usability issues are important (see Table 4).

#### 3.2.2. Overall Usability, Design, and Comfort

Participants' responses to the SUS are presented in Table 5. The average SUS score was 55.83 (SD: 15.37) “ok” ranging from 22.50 “worst imaginable” to 80.00 “good.” Cluster analysis revealed two clusters: cluster 1 (*n* = 8) with a mean SUS score of 67.5 “ok” and cluster 2 (*n* = 7) with a mean SUS score of 42.5 “poor.” Further correlation analysis according to Pearson revealed no statistical significant correlations between the SUS score and participant variables like age (0.114, *p*: 0.687), gender (0.219, *p*: 0.451), years of job experience (0.248, *p*: 0.373), experience with feasibility studies (−0.084, *p*: 0.766), computer skills (0.011, *p*: 0.969), and knowledge in Boolean algebra (−0.196, *p*: 0.483). Additionally formulated items for assessing design and comfort showed that participants on average were positive about the design of the interface (mean: 3.56, SD: 0.814), felt comfortable using the QB in English (mean: 4.38, SD: 0.719), and felt comfortable with the way of building a query (mean: 3.50, SD: 0.730).

In the question regarding what participants appreciated most about the QB they named (a) user-friendliness (*n* = 9), primarily the very intuitive drag and drop interface, the user-friendly terminology, and ease of learning and the layout and (b) functionality of the QB (*n* = 5). With regard to the functionality especially simplified search operators across medical terminology, timeline options for diagnoses, possibility to edit queries, predictive searching capability when finding terms to be included, and data that can be obtained were assessed positively.

Room for improvement may focus on enhancing the user-friendliness by providing more icons (the system is seen as “program” based which can put off nontechnical people) (*n* = 1) and enhancing the user-friendliness of terms (*n* = 1). Furthermore, participants suggested extensively revising the logic of the sequence of adding clinical parameters and timeline parameters (*n* = 3). The options to define time ranges and occurrences should also be reworked (*n* = 3). Additionally, functions to multiselect terminologies and to link two search criteria (because some of them are interdependent upon each other) should be available (*n* = 1). The search items should be provided in a “medical sort” (which most probably was intended to denote “medical sorting order”), not alphabetically (*n* = 1). In addition, the search function should be improved to find the search terms, for example, by a phonetic search, and different ways of searching should be offered (*n* = 2). Further, participants noted that a confirmation notification after updating information in the text field would be helpful (*n* = 1). Other comments were that the constraints can be confusing (when and how to apply them) (*n* = 1) and that disruption can occur if specific content shall be copied (*n* = 1). Besides, the reference number of each criterion should be available for reuse in subsequent inclusion/exclusion criteria (*n* = 1) and it should be referred to criteria within the query rather against the parent search terminology (*n* = 1). Shortcuts (*n* = 1) and a help function were also suggested (*n* = 2).

#### 3.2.3. Quality of Training

Overall, participants were satisfied with the training (mean: 3.53, SD: 0.640) and agreed that the topics were relevant for the tasks (mean: 4.20, SD: 0.414). Furthermore, they stated that the training material was helpful (mean: 4.13, SD: 0.352) and that the content was well organized and easy to follow (mean: 3.93, SD: 0.640). Participants also were positive about the speed of the training video (mean: 3.64, SD: 0.842) and the time allotted for the training (mean: 3.54, SD: 0.877). However, they mostly did not agree to have enough information available (mean: 3.27, SD: 1.033). Regarding the usefulness of the training experience for work, participants were rather neutral (mean: 3.40, SD: 0.737) (see Table 6). Pearson correlation analysis revealed no statistical significant relationships between overall training satisfaction and participants' variables (age: −0.010, *p*: 0.972; gender: 0.230, *p*: 0.428; years of job experience: 0.073, *p*: 0.795; experience with feasibility studies: −0.228, *p*: 0.414; computer skills: 0.066, *p*: 0.815; and knowledge in Boolean algebra: 0.273, *p*: 0.324).

Asked for recommendations to improve the training, participants named (a) optimization of the video with respect to higher resolution, audio instructions, text cues, and reduction of video speed (*n* = 7), (b) provision of clearer instructions (e.g., on the sequencing of questions and how to deal with time occurrences) and more specific details about the queries (e.g., it was not clear what is meant by “first” and “last” in the EHRs in this context) (*n* = 7), (c) improvement of the terminology for sections and provision of more information regarding the terms (*n* = 2), and (d) language enhancements (*n* = 3).

### 3.3. Task Success

The results of the task success analysis (see Table 7) show that testers were able to correctly complete the creation and execution of queries 1 and 2 in ten out of thirteen cases, whereas four out of twelve of the testers could successfully complete query number 3. Two of the testers did not share the screenshots containing the results and other four either did not share one of the queries or the screenshots were insufficient to determine the success of the task completion.

## 4. Discussion

The system users stated that the platform was easy to use and that they were able to perform the tasks for which it was designed with the provided amount of training. There were many positive comments towards functionality and usability. However, the SUS of nearly 56, in the threshold of an “OK” result, suggests that there is still room for improvement. This is also reflected by the free text comments that suggested modifications to the platform usability. According to the feedback given after the completion of the tasks, it seems that for simple and normal queries the system is usable, but for complex ones the users have difficulties (see Tables 3 and 7). It cannot be concluded whether this is due to system deficiencies or insufficient training.

### 4.1. Strengths and Weaknesses of Study

To assure a robust and reliable methodology, this study was preceded by a pretest to assure the technical system functionality and the appropriateness of the methodology.

The different levels of query complexity were designed for a test environment. Only the second query reflected a real feasibility query. The third query was especially designed to test different functionalities and the temporal constraints. This type of query is not likely to be used in a real world scenario. Interestingly this query had not only the highest error rate but also the highest difficulty and lowest satisfaction among the user ratings. The free text comments showed that the provided training was not sufficient for those kinds of queries. It might be possible that the SUS was biased by the difficulty and the inability to successfully complete the task, as the users were asked to complete the SUS questionnaire immediately after finishing the third query.

An alternative evaluation approach would have been to make use of the Thinking Aloud method instead of (or complementing) the SUS, as, for example, in [20]. Since the study was executed at the participant's workplace, this method seemed inappropriate, though. Furthermore, using a standardized and established method that produces a single overall score like the SUS makes the outcome comparable to possible future studies that examine a similar matter.

The test setting was not absolutely the same for all participants since the study was executed at their workplace. Unrecorded distractions might have occurred. However, this was allowed since it reflected a realistic scenario rather than a laboratory setting.

The questionnaire was provided in English only. In [21], Finstad demonstrated that the vocabulary used by the SUS might be hard to understand by nonnative speakers. However, since 75% of the participants were native English speakers (Table 2), the probability of a language induced bias can be considered to be rather low.

The relatively low number of participants (*n* = 16) might be considered a methodological weakness. However, the basic population of suitable domain experts with no knowledge of the platform was already quite low beforehand. Tullis and Stetson [22] showed that a sample size of about 12 participants yields good results for the SUS. Therefore, 16 participants can be considered sufficient. However, the effect of expertise must be taken into consideration with respect to usability testing in general and the SUS in particular. Experienced users in a given domain tend to provide a slightly higher, more favourable SUS score than users with either no or limited experience [23]. The demographic analysis shows that about half of the participants assessed themselves to have much experience with feasibility studies (see Table 2). This might have tampered the SUS score.

### 4.2. Relation to Other Studies

The technical query model that is used to collect the patient cohorts from the distributed data sources [9] and the possibilities and requirements for an actual use of the EHR4CR platform [24] have already been evaluated in earlier studies.

The usability of patient cohort identification systems for the purpose of clinical trial feasibility assessment, however, has only been examined for a few isolated solutions so far. In 2005 [25], the evaluation of a clinical trial alert system that triggered reminders whenever patient data met EC during routine visits is described. A survey comprising 14 questions was used for that study; no standardized method was used and no score was calculated. The evaluation of another site-specific tool named “ASAP” (Advanced Screening for Active Protocols) from the Ohio State University Medical Center was published in 2012 [20]. That study is based upon Thinking Aloud protocols and a survey of 10 questions. The users of the ASAP system were asked to rate the ease of use and the perceived usefulness of the tool for the user's clinical environment on a Likert scale of 1 to 5. Additionally, the users were asked if the tool would be useful for screening patients based on their experience during the usability test. Again, no standardized usability testing and no score calculation took place.

A project with a similar scope like EHR4CR and the FARSITE evaluated the technical results but not the usability [8]. Another project with a complexity comparable to EHR4CR, but with a different focus, is the Cancer Translational Research Informatics Platform (caTRIP) as part of caBIG (Cancer Biomedical Informatics Grid), which allows querying across a number of data services, joining common data elements, and viewing the results. It provides the user with the ability to construct, execute, and share distributed queries in a graphical environment. The user interface is claimed to be user-friendly, but the proof is not provided [26].

Finally, an evaluation of the i2b2 user interface needs to be mentioned, since i2b2 provides a comparable functionality and a comparable query assembling paradigm like the EHR4CR QB. The abstract of that study [27] claims that the usability of i2b2 was evaluated, but it actually rather evaluated the applicability (i.e., if it can be used for a given scenario) than the usability.

In summary it can be stated that, to the authors' knowledge, this investigation is the most systematic usability evaluation, including a usability score, of a cohort identification system for CTs so far.

### 4.3. Meaning and Generalizability

As a qualitative rather than quantitative study, questions of the generalizability of the results rest on the extent to which the selected users can be seen as typical of the eventual desired population of users for the platform. The selection of users for this study came from within the EHR4CR project, meaning that this selection is from precisely the target population of users of the tool. This would indicate that the composition of the study would carry a high degree of situational representativeness. However, due to the qualitative nature of the results it is difficult to justify with certainty the applicability of the results to different sets of users, possibly from different countries and different working environments.

In terms of the characteristics of the study population, half of the users judged their current experience with feasibility studies as “high.” It could be expected that users of the system, when deployed in a real world scenario, would fall more into this category than nonexpert users as such a system would most likely be deployed to complement existing feasibility tools or practices. Given the relatively low number of participants in the study it was not possible to derive any statistically valid results from a comparison of expert and nonexpert users, where perhaps such an analysis of the responses split along these lines would have yielded differences in the subject's view of the system.

The study asked participants to construct three feasibility queries of varying complexity. Of these, the first focused only on the most basic features of the QB portion of the platform whilst the third was a catch-all for the full range of advanced functionality available. Only the second, which was based on the transformation of a real world country feasibility study criteria by experts, could be seen to reflect more accurately the type of criteria that the tool would be used to construct in real world use cases. As such the results of the study, which were derived from feedback from users based on carrying out all three tasks (easy to complex), may not generalize to real world use cases predominately reflected by the type of query.

### 4.4. Future Work/Questions

The EHR4CR project team may consider enhancing the current system utilizing the feedback from the testers presented in this paper. In a future study it should be tested whether users have fewer difficulties with temporal constraints and very complex queries if more specific training is provided to them.

## 5. Conclusions

The user satisfaction of the EHR4CR QB was successfully evaluated with a positive result in a real world, multinational setting. Functionalities of time constraints need to be revisited as they are often part of clinical trials EC. It has been proven that, with a relatively small amount of training, users are able to correctly create and execute simple feasibility queries in the EHR4CR QB. Besides, this evaluation provides a list of features and modifications that such systems should comply to.

## Supplementary Material

The additional file contains the complete set of questions automatically extracted from the original web based questionnaire in pdf format.

## Figures and Tables

**Table 1:** Queries construction.

	Query 1		Query 2		Query 3	
	Criterion	Temporal constraints	Criterion	Temporal constraints	Criterion	Temporal constraints
Inclusion criteria						
Gender	Female	—	—	—	—	—
Age	>50 years	—	>18 years	—	>18 years	—
Diagnosis	—	—	Non-insulin-dependent diabetes mellitus	—	Heart failure	At most 3 years before the query
Lab values	—	—	Body mass index 25 < *X* < 40	—	Left ventricular ejection fraction <40%	At most 14 months before the query
	—	—	Haemoglobin A1c > 7,5%	—	Systolic blood pressure >2,0 mmHg	2 times separated with at least 2 months in between

Exclusion criteria						
Diagnosis	—	—	Acute myocardial infarction	—	Cardiomyopathy in the puerperium	Between 0 and 5 months before the heart failure
	—	—	—	—	Acute myocardial infarction	Between 5 and 30 months before now
	—	—	—	—	Percutaneous coronary intervention	Between 5 and 30 months before now
	—	—	—		Operation on heart	Between 5 and 30 months before now
Treatment	—	—	Using insulin and analogues	—	Vasodilators used in cardiac diseases	—
	—	—	—	—	Phosphodiesterase inhibitors	—
	—	—	—	—	Cardiac stimulants	—

This table shows criteria composing each query. Users had to construct queries following this pattern in order to find the number of patients corresponding to the set of criteria.

**Table 2:** Sample characteristics.

Variable	*n*	%
Current job group		
Feasibility manager	7	43.75
Data manager	1	6.25
Trial manager	2	12.50
Other (e.g., head of clinical operations, enrolment specialist, clinical operations portfolio manager)	6	37.50
Work experience (years)^*∗*^	16	3.01 (1.680)
Gender		
Male	7	43.75
Female	8	50.00
(No answer)	1	6.25
Age (years)^*∗*^	14	43.57 (5.827)
(No answer)	2	
Native language		
English	12	75.00
German/Swiss German	2	12.50
Polish	1	6.25
(No answer)	1	6.25
Difficulties regarding English		
Never, English is my native language	11	66.75
Never, English is not my native language	2	12.50
Rarely	2	12.50
(No answer)	1	6.25
Usage of similar systems in the past		
No	13	81.25
Yes	3	18.75
Experience with feasibility studies		
Little experience	3	18.75
Some experience	4	25.00
Much experience	9	56.25
Computer skills		
Average computer skills	2	12.50
Good computer skills	8	50.00
Excellent computer skills	6	37.50
Knowledge in Boolean algebra		
No knowledge	3	18.75
Little knowledge	4	25.00
Average knowledge	3	18.75
Good knowledge	5	31.25
Excellent knowledge	1	6.25

Characteristics of the participants (*n* = 16). Summarized number and row percentage per category; ^*∗*^for “work experience” and “age” mean and standard deviation were calculated; *n* = 16 participants.

**Table 3:** Perceived task difficulty and satisfaction.

Item	Task 1		Task 2		Task 3		Wilcoxon-Test, *p* value		
	Mean	SD	Mean	SD	Mean	SD	T1-T2	T1-T3	T2-T3
Task difficulty	3.94	0.929	3.75	1.000	2.63	1.088	0.582	0.006^*∗*^	0.005^*∗*^
Satisfaction with the ease of completing the task	3.81	0.911	4.06	0.574	2.75	0.856	0.271	0.007^*∗*^	0.001^*∗*^
Satisfaction with the amount of time it took to complete the task	3.88	0.885	3.75	0.856	2.94	0.680	0.755	0.017^*∗*^	0.005^*∗*^
Satisfaction with the functionality provided	3.69	0.873	3.81	0.655	2.88	0.957	0.557	0.010^*∗*^	0.002^*∗*^

Mean ratings (5-point rating scale), standard deviations, and *p* values of Wilcoxon-Test, ^*∗*^significant at the *p* = 0.05 level; *n* = 16 participants.

**Table 4:** List of usability issues encountered by participants.

Task	Missing functionalities	User number	Expert review
Task 1	Criteria of >49 years were selected but appear as ≥49 years	User 01	Not important; mistake in specification of query not tool
	No ability to execute results for all countries, only for UK/no response when clicking on all countries	User 19	Medium importance; probably a problem with available sites not with the tool
	The query in eclectic format did not show up and looked similar to screen shot in training manual	User 06	Low importance; feature only used for testing probably be removed for “real world” version of tool

Task 2	Than & less selections appear transformed into more & less than OR EQUAL to	User 01	Not important; mistake in specification of query not tool

Task 3	The sequence of building the query is not clear; system seems to require it in reverse (i.e., the parameters of time to be entered before the diagnosis)	User 18, user 19	Important; comment on usability though unspecific
	Entering exclusion criteria (e.g., 3.3.4 EC02) is cumbersome	User 06	Important; comment on usability though unspecific
	No visible option how to add a range of 5–30 months; the range always began at 0 months	User 01	Medium importance; option is there when user selects “between” rather than “more than” or “less than,” user interface issue, or poor documentation
	No way to clear just one component from the query; “clear” clears all components/if you want to change particular part of the inclusion or exclusion criteria, you have to delete the whole; it would be better to delete parts	User 06, user 21	Medium/low; true but the individual inclusion sections are never hugely complex so deleting all is not too bad
	The “before now” button did not work several times	User 19	Important; was not seen this reproduced though
	The run function and eclectic format were not possible; computer crashed when running the query or doing it in eclectic format/“does not compute” message appeared, when trying to generate the eclectic format	User 21, user 01	Important; a “crash” reproduced elsewhere was not seen
	System feedback was that query had been saved, but it does not appear to have been	User 01	Important; true in terms of lack of feedback, but it is always saved
	If you want to check a specific value of a criterion (e.g., if left ventricular ejection fraction was correctly entered and you want to check it later) you are not able to see it by clicking on the symbols	User 21	Important; this information appears at the bottom of the screen and not where user would originally see it and may need to scroll, user interface issue

Responses to the open-ended question “What function or feature do you miss for this task?,” and expert review of usability issues; *n* = 16 participants.

**Table 5:** Results of the System Usability Scale (SUS).

SUS item	*N* (valid)	Mean	SD
I think that I would like to use the Query Builder frequently	16	3.63	0.885
I [did not find] the Query Builder unnecessarily complex^*∗*^	16	3.06	0.929
I thought the Query Builder was easy to use	16	3.38	0.719
I think that I [would not] need assistance to be able to use the Query Builder^*∗*^	16	2.94	0.998
I found the various functions in the Query Builder were well integrated	15	3.07	0.961
I [did not think] there was too much inconsistency in the Query Builder^*∗*^	15	3.33	1.047
I would imagine that most people would learn to use the Query Builder very quickly	16	3.25	1.000
I [did not find] the Query Builder very cumbersome to use^*∗*^	16	3.19	0.834
I felt very confident using the Query Builder	16	3.06	0.854
I [did not need] to learn a lot of things before I could get going with the Query Builder^*∗*^	16	3.00	1.033
Overall SUS score	**15**	**55.86**	**15.37**

Mean rating (5-point scale from 1 “strongly disagree” to 5 “strongly agree”), standard deviations, and overall SUS score. Items marked with an asterisk (^*∗*^) were reverse coded; *n* = 16 participants.

**Table 6:** Quality of the training.

Items	*N* (valid)	Mean	SD
The topics covered by the training were relevant for the tasks	15	4.20	0.414
The time allotted for the training was sufficient	13	3.54	0.877
The content of the training was well organized and easy to follow	15	3.93	0.458
The materials distributed were helpful	15	4.13	0.352
The speed of the training video was appropriate	14	3.64	0.842
The amount of information was sufficient for solving the tasks	15	3.27	1.033
This training experience will be useful in my work	15	3.40	0.737
Overall, I am satisfied with the training	15	3.53	0.640

Mean ratings (5-point scale from 1 “strongly disagree” to 5 “strongly agree”) and standard deviations; *n* = 16 participants.

**Table 7:** Task success.

User ID	Task 1	Task 2	Task 3
User 1	S	S	F
User 2	P	P	
User 3	S	S	F
User 4	F	S	P
User 5		S	F
User 6	S		F
User 7	S	S	S
User 8	S	S	P
User 9	S	S	F
User 10	S	S	S
User 11	S	S	S
User 12	S	P	P
User 13	S	S	S
User 14	F	F	

S: success is given when the whole completion of the task is successful. P: partial success is given when the user commits no more than a severe mistake (wrong use of the EC) or no more than two minor mistakes (wrong use of the temporal constraints). F: failure is given when the user commits more than one severe mistake or more than two minor mistakes.
