# Supplementary material for: User Satisfaction Evaluation of the EHR4CR Query Builder: A Multisite Patient Count Cohort System
Source: Biomed Res Int. 2015 Oct 11;2015:801436. doi: 10.1155/2015/801436 (PMC4619869; doi:10.1155/2015/801436)
Supplement: Supplementary file 1 — The additional file contains the complete set of questions automatically extracted from the original web based questionnaire in pdf format. [file 801436.f1.pdf]

Dear study participant,

The purpose of this questionnaire is to assess your experiences and satisfaction with the user interface (UI) of the Query Builder you have tested and the training you received before.

By completing it, you are enabling us to remedy any shortcomings or weaknesses identified.

The questionnaire consists of four parts:

Section A: Post-Task Assessment: is for assessing your experience with the Query Builder after each task.

Section B: Usability and Acceptance of the Query Builder: is for your overall assessment of the Query Builder after all tasks are completed.

Section C: Suitability of the Training: is for your evaluation of the training you received.

Section D: Background Information : is for collecting demographic data which allows for a scientifically sound interpretation of the test results.

Initial questions and consent

1. If applicable, please check the box to continue.

I declare that I was informed about the purpose of this survey and that I participate voluntarily. ☐

2. Please enter your tester ID

Section A1: Task 1 questions

After completing task 1: For each of the following statements, indicate your answers by marking the appropriate box and filling out the blank space if applicable.

A1.1.

	very difficult	somewhat difficult	neither difficult nor easy	somewhat easy	very easy	cannot be assessed
Overall, this task was:	☐	☐	☐	☐	☐	☐

A1.2.

	strongly disagree	disagree	neither agree or disagree	agree	strongly agree	cannot be assessed
I am satisfied with the ease of completing this task.	☐	☐	☐	☐	☐	☐

A1.3.

	strongly disagree	disagree	neither agree or disagree	agree	strongly agree	cannot be assessed
I am satisfied with the amount of time it took to complete this task.	☐	☐	☐	☐	☐	☐

A1.4.

	strongly disagree	disagree	neither agree or disagree	agree	strongly agree	cannot be assessed
I am satisfied with the functionality provided when completing this task.	☐	☐	☐	☐	☐	☐

A1.5. *If you marked the box for "strongly disagree " or "disagree":* What function or features do you miss for this task?

A1.6. Please take a snapshot of the query generated and upload it here

A1.7. Please take a snapshot of the RESULTS and upload it here

Section A2: Task 2 questions

<u>After completing task 2</u>: For each of the following statements, indicate your answers by marking the appropriate box and filling out the blank space if applicable.

A2.1.

	very difficult	somewhat difficult	neither difficult nor easy	somewhat easy	very easy	cannot be assessed
Overall, this task was:	☐	☐	☐	☐	☐	☐

A2.2.

	strongly disagree	disagree	neither agree or disagree	agree	strongly agree	cannot be assessed
I am satisfied with the ease of completing this task.	☐	☐	☐	☐	☐	☐

A2.3.

	strongly disagree	disagree	neither agree or disagree	agree	strongly agree	cannot be assessed
I am satisfied with the amount of time it took to complete this task.	☐	☐	☐	☐	☐	☐

A2.4.

	strongly disagree	disagree	neither agree or disagree	agree	strongly agree	cannot be assessed
I am satisfied with the functionality provided when completing this task.	☐	☐	☐	☐	☐	☐

A2.5. ***<u>If you marked the box for "strongly disagree " or "disagree":</u>*** **What function or features do you miss for this task?**

A2.6. **Please take a snapshot of the query generated and upload it here**

A2.7. **Please take a snapshot of the RESULTS and upload it here**

Section A3: Task 3 Questions

<u>After completing task 3</u>: For each of the following statements, indicate your answers by marking the appropriate box and filling out the blank space if applicable.

A3.1.

	very difficult	somewhat difficult	neither difficult nor easy	somewhat easy	very easy	cannot be assessed
Overall, this task was:	☐	☐	☐	☐	☐	☐

A3.2.

	strongly disagree	disagree	neither agree or disagree	agree	strongly agree	cannot be assessed
I am satisfied with the ease of completing this task.	☐	☐	☐	☐	☐	☐

A3.3.

	strongly disagree	disagree	neither agree or disagree	agree	strongly agree	cannot be assessed
I am satisfied with the amount of time it took to complete this task.	☐	☐	☐	☐	☐	☐

A3.4.

	strongly disagree	disagree	neither agree or disagree	agree	strongly agree	cannot be assessed
I am satisfied with the functionality provided when completing this task.	☐	☐	☐	☐	☐	☐

A3.5. ***<u>If you marked the box for "strongly disagree " or "disagree":</u>*** **What function or features do you miss for this task?**

A3.6. **Please take a snapshot of the query generated and upload it here**

A3.7. **Please take a snapshot of the RESULTS and upload it here**

Section B: Usability and Acceptance of the Query Builder

B1. **For each of the following statements, mark <u>one</u> box that best describes your reaction to using the interface of the Query Builder *today*.**

	strongly disagree	disagree	neither agree or disagree	agree	strongly agree	cannot be assessed
I think that I would like to use the Query Builder frequently.	☐	☐	☐	☐	☐	☐

	strongly disagree	disagree	neither agree or disagree	agree	strongly agree	cannot be assessed
I found the Query Builder unnecessarily complex.	☐	☐	☐	☐	☐	☐
I thought the Query Builder was easy to use.	☐	☐	☐	☐	☐	☐
I think that I would need assistance to be able to use the Query Builder.	☐	☐	☐	☐	☐	☐
I found the various functions in the Query Builder were well integrated.	☐	☐	☐	☐	☐	☐
I thought there was too much inconsistency in the Query Builder.	☐	☐	☐	☐	☐	☐
I would imagine that most people would learn to use the Query Builder very quickly.	☐	☐	☐	☐	☐	☐
I found the Query Builder very cumbersome to use.	☐	☐	☐	☐	☐	☐
I felt very confident using the Query Builder.	☐	☐	☐	☐	☐	☐
I needed to learn a lot of things before I could get going with the Query Builder.	☐	☐	☐	☐	☐	☐
The user interface of the Query Builder is visually appealing.	☐	☐	☐	☐	☐	☐
I feel comfortable using the Query Builder in English.	☐	☐	☐	☐	☐	☐
I feel comfortable with the way of building a query.	☐	☐	☐	☐	☐	☐

B2. What do you like about the Query Builder?

B3. What do you dislike about the Query Builder?/ What therefore should be improved?

Section C: Suitability of the Training

C1. Please indicate your level of agreement with the statements listed below.

	strongly disagree	disagree	neither agree or disagree	agree	strongly agree	cannot be assessed
The topics covered by the training were relevant for the tasks.	☐	☐	☐	☐	☐	☐
The time allotted for the training was sufficient.	☐	☐	☐	☐	☐	☐
The content of the training was well organized and easy to follow.	☐	☐	☐	☐	☐	☐
The materials distributed were helpful.	☐	☐	☐	☐	☐	☐
The speed of the training video was appropriate.	☐	☐	☐	☐	☐	☐
The amount of information was sufficient for solving the tasks.	☐	☐	☐	☐	☐	☐
This training experience will be useful in my work.	☐	☐	☐	☐	☐	☐
Overall, I am satisfied with the training.	☐	☐	☐	☐	☐	☐

C2. If you have any suggestions, how the training material and the training process can be improved, please note them here:

Section D: Background Information

D1. What is your age? (please specify)

Introduce a number (years)

D2. What is your gender?

Female ☐

Male ☐

D3. What is your native language?

- English ☐
- French ☐
- German / Swiss German ☐
- Other ☐

Other

D4. Do you have any difficulties regarding English?

- never, English is my native language ☐
- never, English is NOT my native language ☐
- rarely ☐
- sometimes ☐
- often ☐
- always ☐

D5. What is your current job group?

- feasibility manager ☐
- data manager ☐
- trial manager ☐
- Other ☐

Other

D6. How long have you been in your current position? (please specify)

Express the result with a number of years (e.g.: 2.5)

D7. How would you rate your experience with feasibility studies for clinical trials?

- little experience ☐
- some experience ☐
- much experience ☐

D8. Have you used other similar systems in the past?

If you select yes, please write the name(s) of the system(s) in the text box.

- Yes ☐
- No ☐

D9. **How would you rate your computer skills?**

- no computer skills ☐
- few computer skills ☐
- average computer skills ☐
- good computer skills ☐
- excellent computer skills ☐

D10. **How would you rate your knowledge in Boolean algebra (i.e. the usage of and/or/negation to construct truth values)?**

- no knowledge ☐
- little knowledge ☐
- average knowledge ☐
- good knowledge ☐
- excellent knowledge ☐

D11. **Are there any other comments you would like to add?**
